# ^1^H-NMR Relaxation of Ferrite Core-Shell Nanoparticles: Evaluation of the Coating Effect

**DOI:** 10.3390/nano13050804

**Published:** 2023-02-22

**Authors:** Francesca Brero, Paolo Arosio, Martin Albino, Davide Cicolari, Margherita Porru, Martina Basini, Manuel Mariani, Claudia Innocenti, Claudio Sangregorio, Francesco Orsini, Alessandro Lascialfari

**Affiliations:** 1 Istituto Nazionale di Fisica Nucleare, Sezione di Pavia, 27100 Pavia, Italy; 2 Dipartimento di Fisica, Università degli Studi di Milano, and INFN, 20133 Milano, Italy; 3 Dipartimento di Chimica, Università degli Studi di Firenze and INSTM, 50019 Sesto Fiorentino, Italy; 4 ICCOM-CNR, 50019 Sesto Fiorentino, Italy; 5 ASST GOM Niguarda, Struttura Complessa Fisica Sanitaria, 20162 Milano, Italy; 6 Dipartimento di Fisica, Università degli Studi di Pavia, 27100 Pavia, Italy; 7 Physics Department, Stockholm University, 114201 Stockholm, Sweden; 8 Istituto Nazionale di Fisica Nucleare, Sezione di Firenze, 50019 Sesto Fiorentino, Italy

**Keywords:** magnetic nanoparticles, contrast agents, NMR, relaxometry, coating, iron oxides

## Abstract

We investigated the effect of different organic coatings on the ^1^H-NMR relaxation properties of ultra-small iron-oxide-based magnetic nanoparticles. The first set of nanoparticles, with a magnetic core diameter $d_{s_{1}}$ = 4.4 ± 0.7 nm, was coated with polyacrylic acid (PAA) and dimercaptosuccinic acid (DMSA), while the second set, $d_{s_{2}}$ = 8.9 ± 0.9 nm, was coated with aminopropylphosphonic acid (APPA) and DMSA. At fixed core diameters but different coatings, magnetization measurements revealed a similar behavior as a function of temperature and field. On the other hand, the ^1^H-NMR longitudinal $r_{1}$ nuclear relaxivity in the frequency range ν = 10 kHz ÷ 300 MHz displayed, for the smallest particles (diameter $d_{s_{1}}$), an intensity and a frequency behavior dependent on the kind of coating, thus indicating different electronic spin dynamics. Conversely, no differences were found in the $r_{1}$ relaxivity of the biggest particles ($d_{s_{2}}$) when the coating was changed. It is concluded that, when the surface to volume ratio, i.e., the surface to bulk spins ratio, increases (smallest nanoparticles), the spin dynamics change significantly, possibly due to the contribution of surface spin dynamics/topology.

## 1. Introduction

The synthesis of an efficient Magnetic Resonance Imaging (MRI) contrast agent (CA) requires a full comprehension of the origin of its magnetic static and dynamic properties, so that the chemico-physical microscopic characteristics can be tailored to increase the image contrast. Generally speaking, the MRI image contrast depends on the proton density (i.e., the number of hydrogen nuclei in the interested region per unit volume), the apparent diffusion coefficient *D*, the nuclear longitudinal *spin-lattice* relaxation time $T_{1}$, and the nuclear transverse *spin–spin* relaxation time $T_{2}$, with the last ones strongly affected by the magnetic properties of the optionally introduced CA [1]. Among the possible CAs, iron oxide-based nanoparticles (NPs) are largely used as they exhibit a strong intrinsic magnetization [2] that induces high local field inhomogeneities: this occurrence accelerates both the return to the thermal equilibrium state and the dephasing of proton spins, consequently causing a shortening of the nuclear relaxation times in the “NP-uptake” region and a contrast enhancement in the MRI image [3,4,5,6,7,8,9]. The ability to decrease $T_{1}$ and $T_{2}$ could depend on several characteristics of the NPs, such as magnetic core size, composition, shape, biocompatible coating, etc. [10,11,12,13,14,15,16]). Most of these properties have been thoroughly investigated in recent decades, to find the best candidates for contrast enhancement. On the contrary, few papers studied the coating effect, which, in principle, could impact, to different extents, the biocompatibility [17,18,19,20,21], the biodistribution [22,23,24], the magnetic properties [25,26,27,28], and also the nuclear relaxivity, i.e., the efficacy in decreasing the relaxation times of the nuclei in the region reached by NPs. For in vivo applications, the organic coating can be tailored in different ways, such as by using polymers, sugars, micelles, and other kinds of chemical moieties, and by adding fluorophores, antibodies, drugs or by using magnetic and/or porous materials [29,30,31]. In this regard, it’s worth mentioning that DMSA, PAA and APPA are among the most common moieties employed for coating purposes, as they guarantee colloidal stability and biocompatibility [29,30,31]. All these chemical modifications can significantly impact the relaxation of water protons’ nuclear spins, through the diffusion mechanism, the hydration number, the kind of hydrogen binding [32], the electronic spin value and dynamics and so forth. Moreover, it has to be taken into account that the species, chain length, density and functional group of organic ligands, as well as the thickness of the shells can change the distance of the water protons to the magnetic core, thus varying the nuclear relaxation and, as a consequence, the contrast. Finally, it is worth remembering that a modification of the magnetic nanoparticles (MNPs) coating possibly generates a different surface spins disorder, inducing a local increase of the magnetic field inhomogeneities and/or a higher/lower MNP magnetization, which in turn leads to different $T_{1}$ and $T_{2}$ contrast effects.

In the literature, there are few examples of MRI contrast investigations when the coating is varied. For example, Ahmadpoor et al. [16], by using a 3 Tesla MRI scanner, showed that dopamine–PMA–PEG-coated IONPs (iron oxide-nanoparticles), with a core diameter of 6, 15 and 18 nm, have in all cases higher (up to three times for the 6 nm set, when coating is introduced) $r_{2}$ nuclear relaxivity than PMA–DDA-coated IONPs. LaConte et al.’s results [33] indicate the important role that coating thickness plays in determining the relaxivity of superparamagnetic NPs. Their study on monocrystalline superparamagnetic NPs (6.6 nm core diameter) with coatings of DSPE-PEG of different molecular weights underlined that the transverse relaxivity $r_{2}$ decreases (∼52%) as the molecular weight of the PEG portion of the phospholipid-PEG coating increases, while, on the contrary, $r_{1}$ values increase (∼50%). As concerns the magnetic properties (that also affect ^1^H-NMR relaxation), a recent literature review on this topic (Abdolrahimi et al. [25]) pointed out that a modification of the surface disorder produced by the organic coating, that could be given by the nature of the bonds between the organic molecules and the surface cations, provides a higher MNP magnetization. Additionally, Costo et al. [34] showed that the coating layer increases (by about 10%) the saturation magnetization of 3 nm MNPs. Remarkably, in a previous paper [35], our group investigated the coating effect on the ^1^H-NMR relaxation properties of iron oxide magnetic nanoparticles with a core of 17 and 8 nm diameter: the type of coating investigated was not evidently influencing the longitudinal and transverse relaxometric properties.

Following the above reported state of the art, in this paper we attempt to detect possible effects of the surface spin disorder on the ^1^H-NMR nuclear relaxation times in ferrite core-shell NPs of small (*d* < 9 nm) core diameter. We investigated two sets of MNPs with smaller (two different coatings) and larger (two different coatings, one in common with the smallest MNPs) core diameter, $d_{s_{1}}\approx$ 4.4 nm and $d_{s_{2}}\approx$ 8.9 nm, respectively. The aim being to see the effect of an increased surface to volume (S/V) ratio, i.e., an increased ratio of surface to bulk spins, a situation occurring in the smallest particles. As it will be shown, for a higher surface to volume ratio S/V (core diameter $d_{s_{1}}$) the ^1^H-NMR relaxation times change significantly when the shell is varied, while for lower S/V (core diameter $d_{s_{2}}$) no detectable differences are observed, the last results being in agreement with previously published data [35].

## 2. Materials and Methods

### 2.1. Synthesis of Magnetic Nanoparticles

We investigated two different sets of samples: (i) the first set is made of spherical γ-Fe_2_O_3_ (maghemite) based MNPs with $d_{s_{1}}\approx$ 4.4 ± 0.7 nm mean core diameter (4.4@OA), coated with polyacrylic acid (PAA) (4.4@PAA) or meso-2,3-dimercaptosuccinic acid (DMSA) (4.4@DMSA); (ii) the second one consisting of spherical γ-Fe_2_O_3_ (maghemite) based MNPs with $d_{s_{2}}\approx$ 8.9 ± 0.9 nm mean core diameter (8.9@OA), coated with 3-aminopropylphosphonic acid (APPA) (8.9@APAA) or DMSA (8.9@DMSA).

**Figure 1:**
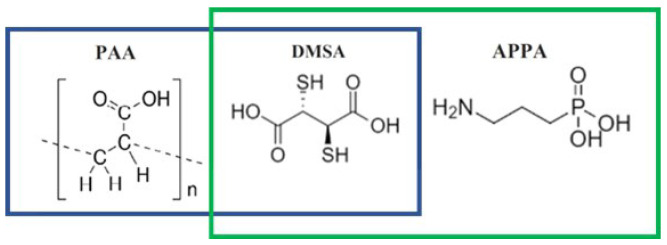
Structure of the different coatings used: PAA, DMSA, APPA. In the Figure, the two sets of samples coatings are also indicated: in the blue box, the coatings used for the smallest MNPs ($d_{s_{1}}\approx$ 4.4 nm); in the green box, the coatings used for the biggest MNPs ($d_{s_{2}}\approx$ 8.9 nm).

The MNPs were synthesized by thermal decomposition of iron acetylacetonate (Fe(acac)_3_), in high-boiling solvent (benzyl ether) in the presence of surfactants (oleic acid, OA; oleylamine, OAM; 1,2 hexadecanediol, HDD), which has emerged as extremely effective in the formation of MNPs with excellent results in terms of size distribution, morphology and crystallinity. Since the synthesized MNPs had a hydrophobic surface coating (oleic acid), it was necessary to modify the surface to make them stable in a physiological environment. The functionalization was carried out by ligand exchange with 3-aminopropylphosphonic acid (APPA), meso-2,3-dimercaptosuccinic acid (DMSA) or polyacrylic acid (PAA; Mw = 1800). The structures of the coatings are reported in Figure 1.

*Synthesis of 4.4-nm iron oxide-based MNPs.* A mixture of Fe(acac)_3_ (0.7064 g, 2 mmol), OAM (2.14 g, 8 mmol), OA (2.26 g, 8 mmol), HDD (1.03 g, 4 mmol), and benzyl ether (50 mL) was magnetically stirred under a flow of nitrogen in a 100 mL three-neck round-bottom flask for 15 min. The resulting composite was heated up to ∼280 °C to reflux with a rate of 8.5 °C/min, and it was kept at this temperature for 15 min under the cover of nitrogen and vigorous stirring. The resulting black-brown mixture was cooled down at room temperature. Then, 60 mL of ethanol were added to induce the precipitation of a black powder. The product was isolated through magnetic separation with a permanent magnet, washed several times with ethanol and finally re-dispersed in toluene (batch 4.4@OA).

*Synthesis of 8.9-nm iron oxide-based MNPs.* A mixture of Fe(acac)_3_ (2.83 g, 8 mmol), OAM (8.56 g, 32 mmol), OA (9.04 g, 32 mmol) and benzyl ether (80 mL) was magnetically stirred under a flow of nitrogen in a 250 mL three-neck round-bottom flask for 15 min. The resulting composite was heated up to ∼290 °C to reflux with a rate of 25 °C/min, and it was kept at this temperature for 90 min under the cover of nitrogen and vigorous stirring. The resulting black-brown mixture was cooled down at room temperature. Then, 60 mL of ethanol was added to induce the precipitation of a black powder. Using a permanent magnet, the product was washed several times with ethanol and finally re-dispersed in toluene (batch 8.9@OA).

*Phase transfer by ligand-exchange with DMSA/PAA.* Iron oxide-based MNPs (25 mg) were dispersed in toluene (5 mL), added to a solution of DMSA/PAA (25 mg) in dimethyl sulfoxide (DMSO, 1 mL)/tetrahydrofuran (THF, 1 mL), sonicated for 1 h and finally incubated at room temperature for 12 h in a mechanical rotating agitator. The precipitate was isolated by magnetic separation using a permanent magnet. First, it was washed several times with DMSO/THF and then with ethanol, and finally re-dispersed in milliQ water (10 mL). Sodium hydroxide was added to the suspension to move the pH to 10, then the pH was adjusted to 7.4 with hydrochloric acid to make it stable (samples 4.4@DMSA, 4.4@PAA and 8.9@DMSA).

*Phase transfer by ligand-exchange with APPA.* Iron oxide-based MNPs (25 mg) were dispersed in CHCl_3_ (5 mL), added to a solution of APPA (25 mg) in a mixture of 2-propanol (1 mL) and tetramethylammonium hydroxide (TMAOH; 25% *w*/*w* aqueous solution, 1 mL), sonicated for 1 h and finally incubated at room temperature for 12 h in a mechanical rotating agitator. The precipitate was isolated by magnetic separation using a permanent magnet. First, it was washed several times with toluene and then with ethanol, and finally re-dispersed in milliQ water (10 mL), obtaining a stable dispersion (sample 8.9@APPA).

### 2.2. Morpho-Dimensional Characterization

Powder X-ray Diffraction (XRD) measurements were carried out to determine the iron oxide phases and the crystal sizes using a Bruker D8 Advance diffractometer equipped with Cu-K*α* (1.54178 Å) radiation and operating in θ-θ Bragg–Brentano geometry at 40 kV and 40 mA, covering a [25°;70°] range, using 0.02° size step and acquisition time of 1 s/step. A CM12 PHILIPS Transmission Electron Microscope (TEM) operating at 100 kV using an LaF6 source was used to assess the MNPs core sizes and shapes. A dilute toluene dispersion of MNPs was drop-casted onto 200 mesh carbon-coated copper grids to prepare the samples. The recorded micrographs were processed by the iTEM Imaging. Platform software (Olympus), and were further analysed with the FIJI open software. The mean diameter and size distribution of the sample was obtained from a statistical analysis over 200 MNPs.

### 2.3. Magnetic Measurements

The DC magnetic measurements were carried out by a SQUID magnetometer (MPMS by Quantum Design Ltd., San Diego, CA, USA) operating within a temperature range of 2 K and 300 K and in the [−5, +5] Tesla magnetic field ($\mu_{0}H$) range. Zero Field Cooled/Field Cooled magnetization curves vs. temperature were acquired in a 5 mTesla probe magnetic field after cooling the sample without (ZFC) and with (FC) the applied field. The magnetization curves *M* vs. *H* were acquired at 5 and 300 K (first set) and 2.5, 250, and 300 (second set), with $\mu_{0}H$ ranging from −5 Tesla to 5 Tesla.

### 2.4. ^1^H Nuclear Magnetic Resonance Measurements

The ^1^H-NMR relaxation times (from which nuclear relaxivites were calculated, see Section 3.4) were measured at different Larmor frequencies of the investigated nuclei, from 10 kHz up to 298 MHz for the first set and from 10 kHz up to 57 MHz for the second one. In particular: (i) for 0.01 MHz <ν< 7.2 MHz we used a Fast-Field-Cycling [36] NMR SMARTracer Relaxometer (Stelar s.r.l., Mede, Italy) with Saturation Recovery (for $T_{1}$), and Spin-Echo (for $T_{2}$) pulse sequences, pre-polarized below 3.7 MHz, and non pre-polarized above 3.7 MHz; (ii) for 7.2 MHz < ν< 57 MHz: we used, for the second set, a Stelar Spinmaster Fourier transform NMR spectrometer, with standard Saturation Recovery ($T_{1}$) and Carr–Purcell–Meiboom–Gill ($T_{2}$) pulse sequences, while for the first one we used a Tecmag Apollo Fourier transform NMR spectrometer with the same pulse sequences in the same frequency range; (iii) for ν> 57 MHz: we used a Tecmag Apollo Fourier transform NMR spectrometer using standard Saturation Recovery ($T_{1}$) and Carr–Purcell–Meiboom–Gill ($T_{2}$) pulse sequences. All measurements were done at room temperature; the samples were prepared by diluting the MNPs in water at a millimolar iron concentration that, from the AAS analysis, resulted: for 8.9@APPA 0.15 mM, for 8.9@DMSA 0.20 mM, for 4.4@DMSA 0.12 mM, and for 4.5@PAA 0.09 mM.

## 3. Results and Discussion

### 3.1. X-rays Diffractometry

**Figure 2:**
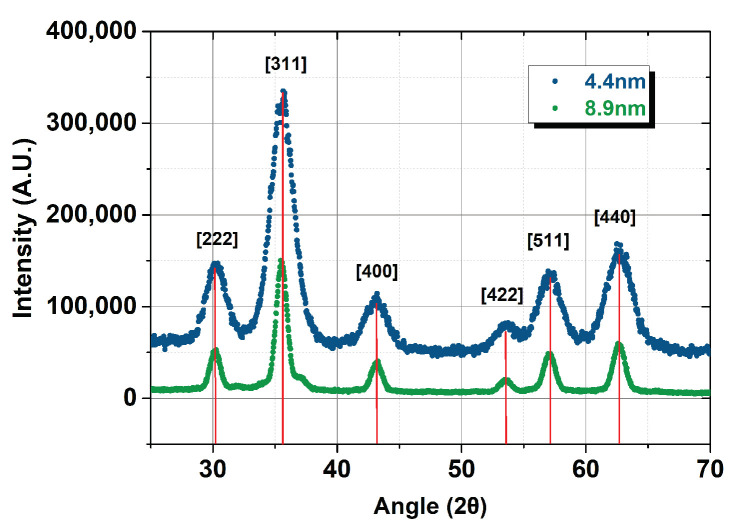
XRD patterns of the 4.4 nm and 8.9 nm samples (powders with oleic acid).

The XRD patterns reported in Figure 2 show the cubic spinel crystallographic structure of the samples, compatible with the presence of both magnetite and maghemite, without other crystallographic phases. The peak width can not allow a more accurate phase attribution by the lattice parameter.

### 3.2. TEM Microscopy

**Figure 3:**
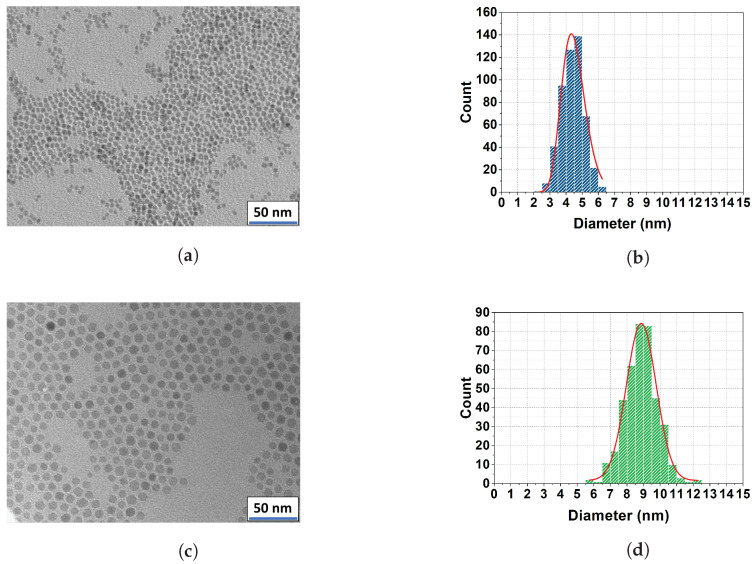
TEM images (**a**,**c**) and histograms (**b**,**d**) reporting the distribution of the core sizes for the first (**a**,**b**) and the second (**c**,**d**) set of nanoparticles coated with oleic acid. The distribution is fitted to a log-normal function; the mean value with the standard deviation are 4.4 ± 0.7 nm and 8.9 ± 0.9 nm, respectively.

The mean diameter and size distribution of the magnetic core for the two sets of samples were obtained from a statistical analysis of images including over 200 MNPs, extracted from TEM measurements. In Figure 3, the TEM images of the samples coated with oleic acid and the relative histograms of the distribution of the magnetic core diameter are reported. The mean ± SD values on histograms were found with the log-normal fit and resulted to be $d_{s_{1}}=4.4\pm 0.7$ nm and $d_{s_{2}}=8.9\pm 0.9$ nm.

### 3.3. Magnetic Measurements

**Figure 4:**
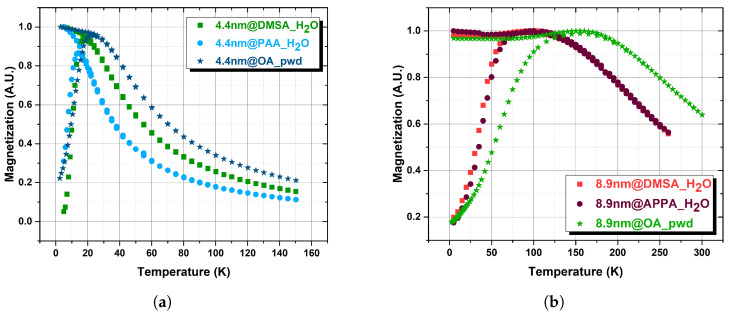
Zero Field Cooled-Field Cooled magnetization of the 4.4 nm (**a**) and 8.9 nm (**b**) set of MNPs collected in a magnetic field $\mu_{0}H$ = 5 mTesla; _pwd stands for powders and _H_2_O for samples in water dispersion.

From the ZFC-FC curves (see Figure 4), we estimated the so-called blocking temperature $T_{B}$ of the samples (corresponding roughly to the peak of the ZFC curve, see Table 1) that separates the blocked regime from the superparamagnetic one (generally speaking, due also to the distribution of the samples size, the blocking temperature does not correspond to the peak of the ZFC curve; however, we decided to use the terminology diffused in literature). It is worth noting that this $T_{B}$ estimation suffers from a number of uncertainties and that to give an absolute reliable value more accurate measurements are necessary (see, e.g., [37,38]). It could be observed that, as the dipolar interactions between MNPs in powder are more intense than those in solution, they have higher blocking temperature values.

**Figure 5:**
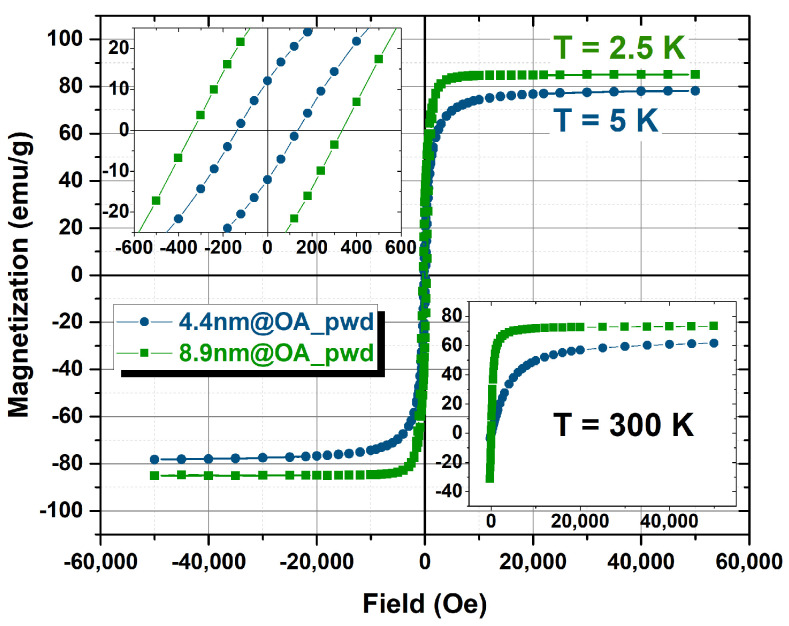
Main figure: hysteresis loops of the different set of powder samples acquired at 5 K for the 4.4 nm samples and 2.5 K for the 8.9 nm samples. The inset on the top-left displays the detail of the hysteresis at low-fields (Magnetization (emu/g) vs. Field (Oe)). The inset on the bottom-right displays the magnetization curves of the powder samples acquired at 300 K (Magnetization (emu/g) vs. Field (Oe)).

**Figure 6:**
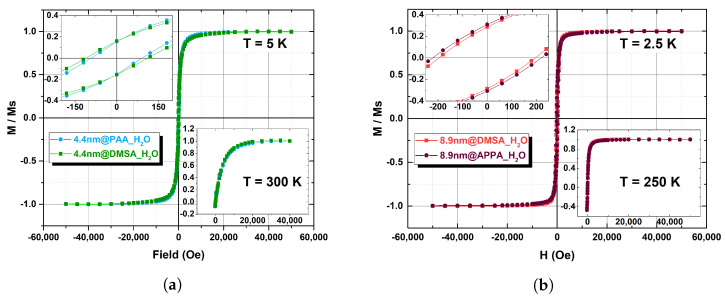
Main figure: hysteresis loops at low temperature of the coated samples of the 4.4 nm set (**a**) and of the 8.9 nm set (**b**), dispersed in water. The insets on the top-left display the detail of the hysteresis at low-field ($M/M_{S}$ vs. Field (Oe)). Each figure shows in the bottom-right inset the corresponding magnetization curves acquired at high temperature ($M/M_{S}$ vs. Field (Oe)).

To assess the saturation magnetization ($M_{S}$) of the magnetic cores of the two sets of samples, the *M* vs. *H* curves were measured on the powder@OA samples, for which the diamagnetic contribution can be neglected. The percentage of OA was evaluated by CHN analysis (12.7% for 8.9 nm and 27% for 4.4 nm) and the magnetization curves obtained for the magnetic component at high and low temperature are shown in Figure 5. $M_{S}$ values, reported in Table 2, have been evaluated from the magnetization curves by fitting the series expansion of the Langevin function at high fields. In Figure 6, the *M* vs. *H* curves at low and high temperature for the samples dispersed in solution are reported for the two sets of core sizes. Conversely to the powder samples, the diamagnetic contribution of the solvent cannot be neglected and to account for this, a linear contribution was removed from the original data. For an effective comparison of the shape of the magnetization curves in each set of samples, the magnetization was then normalized to the corresponding maximum values. As shown in Figure 6, the curves are almost superimposable, suggesting that the effect of the different coating on the magnetic behavior is negligible for both sets. The saturation magnetization of 4.4 nm samples in dispersion at 300 K has been estimated from NMR data fitting (see next paragraph), taking also into account the small variation of $M_{S}$ (within about 10%) reported in the literature [25,34] with respect to powder samples. For 8.9 nm materials, at 300 K we obtained $M_{S}$ = 78 ± 4 emu/g for APPA coated powder samples (data not shown), a value assumed also for 8.9@DMSA sample (from literature [39], for this diameter value, the variation due to coating is reported to be within 5%).

### 3.4. NMR Relaxometry

The MNPs spin dynamics at room temperature has been probed indirectly by means of ^1^H-NMR relaxometry vs. frequency. In fact, it is worth remembering that the longitudinal ($T_{1}$) and the transverse nuclear relaxation ($T_{2}$) enable exploration of the local electron spin dynamics and assessment of the MRI contrast efficiency of MNPs through calculation of the nuclear relaxivity (see Equation (1)). The behaviour of the longitudinal and transverse relaxivity was studied as a function of the proton resonance frequency (Larmor frequency), thus determining the so-called Nuclear Magnetic Relaxation Dispersion (NMRD) profile.

We define the nuclear longitudinal (*i* = 1) and transversal (*i* = 2) nuclear relaxivity $r_{i}$ as:

(1) $$r_{i}=\frac{1}{c}\cdot \left(\frac{1}{T_{i,m}}-\frac{1}{T_{i,dia}}\right)$$

where *c* is the concentration of the paramagnetic centers (usually expressed in mM), 1/T${}_{i,}$${}_{m}$ the measured relaxation rates, and 1/T${}_{i,}$${}_{dia}$ the relaxation rates in the presence of the dispersant (diamagnetic, *dia*) only. As previously mentioned, the *r*${}_{i}$ in Equation (1) quantify the shortening of the nuclear relaxation times $T_{1}$ and $T_{2}$ in the presence of one or more paramagnetic centers with concentration *c* with respect to the case of dispersant only, i.e., the increased capacity of image contrast [5].

**Figure 7:**
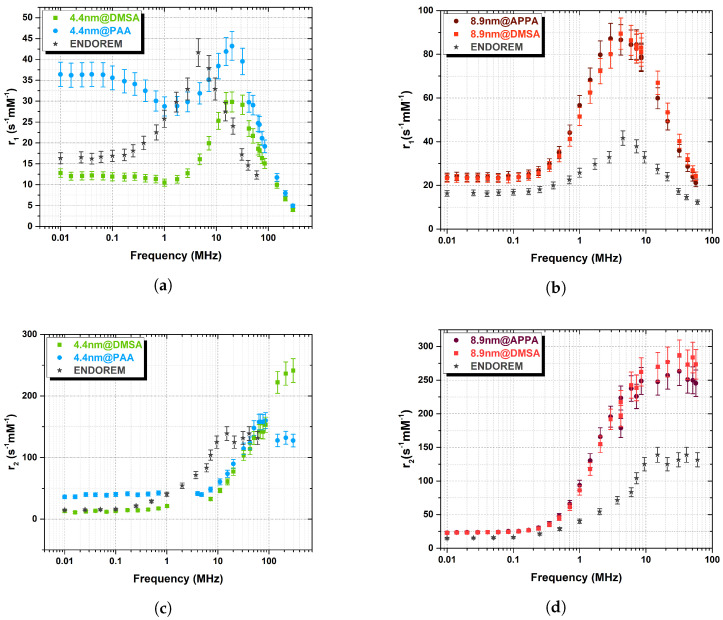
Longitudinal (**a**,**b**) and transverse (**c**,**d**) relaxivity ^1^H-NMR profiles of (**a**,**c**) 4.4 nm samples (coated with DMSA and PAA), and of (**b**,**d**) 8.9 nm ones (coated with DMSA and APPA), collected at room temperature. Gray stars represent the Endorem values. The error bar is calculated a-priori to be 8% (see main text).

As the magnetic core static and dynamic behavior is mostly responsible for the relaxation of the water nuclear magnetization, to study the effect of the coating on the relaxation properties, each set of MNPs was obtained from the same batch of cores but coated with different organic polymers. As can be seen from Figure 7b, within the experimental error, the set of largest particles did not show any relevant difference between the longitudinal NMRD profile of the particles coated with DMSA and APPA and, additionally, no “dispersion” was observed at low frequencies, probably due to the high value of the anisotropy energy. Regarding the smallest set of samples, differences in the peak intensity and in the frequency behaviour are clearly singled out (Figure 7a): the samples coated with DMSA and PAA have a different level of dispersion at frequencies below 10 MHz, and 4.4@DMSA shows a lower peak (about 30%) of the $r_{1}$ relaxivity. These differences might be attributed to different interactions occurring between the coating and the surface spins of the magnetic cores and/or to different surface spins topology; they can be detected only in the smallest set of samples because of the increased surface-to-volume ratio.

Regarding the transverse relaxivity, the frequency behaviour of both sets is qualitatively the same, being almost identical for the biggest particles. It is worth noting that the NMRD profiles of the commercial CA Endorem (SPIO aggregates, coated by a thin dextran layer, with average crystal size of 5.7 nm ($d_{TEM}$) and average hydrodynamic diameter ($d_{DLS}$) of 292 nm [6]), as shown in Figure 7b,d (8.9 nm samples) display a lower relaxivity value, thus suggesting that our 8.9 nm samples are more efficient CA over the whole frequency range, while our 4.4 nm samples are more efficient (than Endorem) above 100 MHz (high fields). The error of the measurements (8%) has been estimated a priori by evaluating the uncertainties coming from different choices of the acquisition parameters, the experimental instrumentation (with particular regard to electronics) and the pulse sequences.

**Figure 8:**
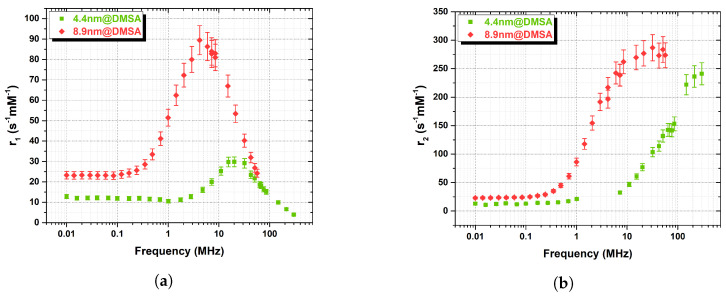
Comparison among the ^1^H-NMRD relaxation profiles of the 4.4@DMSA (green squares) and the 8.9@DMSA (red diamonds) longitudinal (**a**) and transverse (**b**) samples.

Some considerations can be made by comparing the longitudinal relaxivity of particles coated by the same polymer (DMSA) but with different core size (see Figure 8a). Different diameters lead to different $r_{1}$ peak positions and intensities, as theory also predicts [2]. Moreover, it can be noticed how the “dispersion” level (i.e., the “hole” in the curve at low ν) is increased for the smallest core: the anisotropy energy is proportional to the volume [40], i.e., a lower barrier results in a lower $r_{1}$. The transverse relaxivity $r_{2}$ (Figure 8b) for the biggest particles starts to increase at frequencies lower than the smallest particles, with a higher slope; thus, the plateau is promptly reached over 10 MHz, while for the smallest set, the $r_{2}$ is still increasing at 298 MHz.

**Figure 9:**
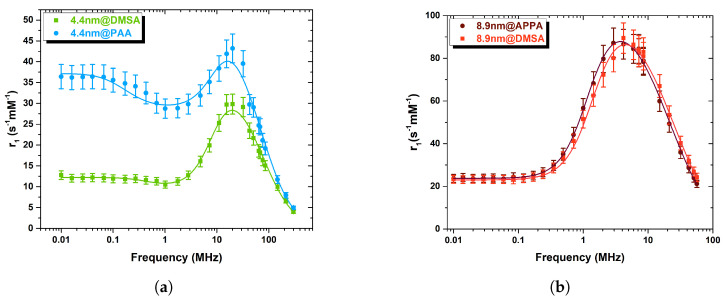
Fit to the heuristic model of Roch-Müller-Gillis of the longitudinal relaxivity ^1^H-NMRD profiles of (**a**) 4.4 nm samples (coated with DMSA and PAA), and of (**b**) 8.9 nm ones (coated with DMSA and APPA); the data have been collected at room temperature.

The $r_{1}$ profiles were then fitted to the heuristic model of Roch–Müller–Gillis [41] (Figure 9) to obtain further information, about the chemico-physical properties of the MNPs, such as the core size *r_C_*, the Néel relaxation time $\tau_{N}$, the minimum approach distance *R* between the magnetic centers and the water molecules, and the anisotropy by means of the free parameter *P*.

Some important points about the initialization of different fitting parameters must be stated. As for the saturation magnetization value of the dispersion: (i) for the smallest particles, $M_{S}$ in dispersion was estimated from the magnetic characterization of the powder sample at room temperature (i.e., 65 emu/g, see Section 3.3), with the constraint of a ± 10% variation; this assumption is justified on the basis of literature results [16,34] and by the small differences that can be induced by different diamagnetic coatings; (ii) for the biggest particles (both 8.9@DMSA and 8.9@APPA), $M_{S}$ was fixed at the value measured at room temperature for 8.9@APPA powders; in fact, remarkably, the coating introduction in our 8.9@APPA particles seems not to significantly affect the saturation magnetization value (5% of increase), in accordance with the literature [39], where a 2% variation of $M_{S}$ between coated and uncoated iron oxide MNPs of about this size was observed.

For both sets the core radius was initially set to the values obtained from the TEM analysis, temperature *T* was set to 300 K, the diffusion coefficient *D* to the theoretical value of 2.3 × 10^−9^ m^2^s^−1^, and the parameter *P*, that quantifies the magnetic anisotropy of the system (*P* = 1 means low anisotropy and *P* = 0 high anisotropy), was allowed to vary between 0 and 1.

In Table 3 the parameter values estimated by the fit are listed.

It is noted that: (i) the $M_{S}$ value for 4.4 nm MNPs saturates at the highest (72 emu/g) imposed constraint; (ii) the distance of minimum approach, *R*, obtains results slightly greater than the core dimension *r_C_* or *r_TEM_*, thus suggesting that the water molecules (whose hydrogen nuclei constitute our local NMR probe) do not penetrate or only partially penetrate the coating layer; (iii) the Néel relaxation time $\tau_{N}$ tends to increase with the anisotropy energy, according to the Arrhenius (or Vogel–Fulcher) law; however, while the Néel relaxation time of the biggest samples is longer than the one of the smallest 4.4@DMSA, the 4.4@PAA sample, despite a higher level of $r_{1}$ dispersion at low frequencies, noticeably displays the greatest $\tau_{N}$; (iv) the parameter *P* resulted lower for the biggest particles than for the smallest ones, a mark of a higher value of anisotropy; moreover the *p* value resulted only slightly higher for the 4.4@PAA sample (0.32) with respect to that of the 4.4@DMSA one (0.27).

The main result, i.e., the different peak intensity and frequency behaviour of 4.4 nm MNPs with different coatings (Figure 7a and Figure 9a), are suggested to be related to the following reasons: (i) slightly different saturation magnetization values ($r_{1max}∼M_{S}^{2}\cdot D/r_{c}^{2}$); (ii) different *p* value which originates from different magnetic anisotropy, meaning diverse core volume and energy barrier; (iii) different $\tau_{N}$, as resulting from the fit; this is justified by possible variations of $\tau_{0}$ and of the magnetic anisotropy when the coating is changed, i.e., different spin topology or dynamics at the MNP’s core surface.

## 4. Conclusions

The effect of the coating on the spin dynamics and the MRI image contrast capability of iron-oxide-based core-shell compounds has been investigated. Two sets of colloidal dispersion of MNPs have been studied. The first set is constituted of two samples with a ferrite core diameter $d_{s_{1}}$ = 4.4 nm and coated with different polymeric biocompatible moieties, PAA and DMSA. The second set is made of two 8.9 nm core-sized samples, coated with APPA and DMSA.

The morpho-structural and magnetic characterizations showed a superparamagnetic behaviour for all samples that display a blocking temperature below 300 K. The ^1^H-NMR $T_{1}$ and $T_{2}$ relaxation times measurements (from which longitudinal $r_{1}$ and transverse $r_{2}$ relaxivity NMRD frequency profiles were estimated), were performed in the range 10 kHz ÷ 298 MHz for the first set and 10 kHz ÷ 57 MHz for the second one. The profiles display the typical frequency behavior of superparamagnetic nanoparticles with *d* < 20 nm, with a dispersion at low frequency for the smallest particles. Within the experimental error, one can observe the following: (i) both $r_{1}$ and $r_{2}$ show no difference for 8.9 nm samples with different coatings; (ii) the smallest 4.4 nm set displays NMRD profiles with significant differences in the $r_{1}$ intensity and frequency behaviour among 4.4@DMSA and 4.4@PAA. In particular, we noticed that the intensity of the 4.4@DMSA peak occurring at about 20 MHz is smaller (by about 30%) than the one of 4.4@PAA and the degree of $r_{1}$ dispersion at low frequencies is higher for 4.4@PAA, a signature of possible different anisotropy energy; (iii) comparing particles with the same DMSA polymeric coating but different core size, we observed that (a) the maximum values of $r_{1}$ and $r_{2}$ relaxivities are higher for the biggest set, as expected from the larger anisotropy and saturation magnetization; (b) for the smallest MNPs, a shift toward higher frequencies (ν > 10 MHz) of the $r_{1}$ peak position and of the $r_{2}$ plateau is present.

The longitudinal experimental ^1^H-NMR relaxation data were then fitted to the heuristic model of Roch–Müller–Gillis. Concerning the parameters estimated by the model it is noted that: (a) the Néel relaxation time ranges from 7.2 × 10${}^{-10}$ to 3.4 × 10${}^{-9}$ s; (b) the resulting minimum approach distances were slightly bigger than the core radii estimated by the fit, thus suggesting that the surrounding water molecules do not penetrate (or only partially penetrate) the coating; (c) the *P* parameter of the biggest samples resulted lower (close to zero) than the smallest one, highlighting their higher magnetic anisotropy.

Remarkably, observing the difference in $r_{1}$ intensity and frequency behaviour among 4.4@DMSA and 4.4@PAA, we suggest that the different coatings in small MNPs could play a crucial role in the nuclear relaxation, determined by the (electronic) spin dynamics. Thus, we suggest that in the smallest set of samples, due to the higher surface-to-volume ratio, the surface spin dynamics and/or the spin topology are affected by the polymeric layer, whose “interaction” with the core surface spins might influence the magnetic dynamics of the MNPs.

In conclusion, as advances on the state of the art, we found that the kind of organic coating should be taken into account to optimize the magnetic nanostructures for MRI (and MFH—magnetic fluid hyperthermia) applications, not only for its relevance in terms of stabilization, functionalization and biocompatibility, but also for its possible role in tuning the relaxation dynamics and thus the applicative efficiency. Moreover, our experimental data open the way to more deeply investigate, using NMR, the surface spin dynamics in MNPs, which are still mostly unknown. 

## Figures and Tables

**Table 1:** Blocking temperature $T_{B}$ of the first and second set of MNPs.

Sample	*T_B_* (K)
4.4@OA_pwd	22 ± 9
4.4@PAA_H_2_O	14 ± 5
4.4@DMSA_H_2_O	18 ± 6
8.9@OA_pwd	155 ± 58
8.9@APPA_pwd	195 ± 75
8.9@APPA_H_2_O	100 ± 53
8.9@DMSA_H_2_O	95 ± 53

**Table 2:** Saturation magnetization $M_{S}$ of the first and the second set of MNPs powders at low (5 K and 2.5 K for the first and the second set, respectively) and high temperature (300 K).

Sample	$M_{S,\;\mathrm{low}\;\mathrm{temperature}}$ (emu/g)	$M_{S,\;\mathrm{high}\;\mathrm{temperature}}$ (emu/g)
4.4@OA_pwd	79 ± 5	65 ± 4
8.9@OA_pwd	85 ± 5	74 ± 4

**Table 3:** Parameters obtained from the fit of the $r_{1}$ NMRD profiles. The Néel relaxation time $\tau_{N}$, the minimum approach distance *R*, and the parameter *P* were evaluated from the fit. (See the main text for more details).

Sample	$M_{S}$	$r_{\mathrm{TEM}}$	$r_{C}$	τ ${}_{N}$	*R*	*P*
	(emu/g)	(nm)	(nm)	(ns)	(nm)	
4.4@PAA	72 ± 7	2.20 ± 0.35	2.9 ± 0.2	3.4 ± 0.5	3.9 ± 0.3	0.32
4.4@DMSA	72 ± 3	2.20 ± 0.35	3.1 ± 0.3	0.72 ± 0.10	3.8 ± 0.5	0.27
8.9@DMSA	78	4.45 ± 0.45	5.7 ± 0.6	1.3 ± 0.3	6.1 ± 0.6	0
8.9@APPA	78	4.45 ± 0.45	6.1 ± 0.7	1.5 ± 0.3	6.5 ± 0.6	0.02

## Data Availability

The authors confirm that the experimental data supporting the findings of this study are available within the article.
